# A 300-kb microduplication of 7q36.3 in a patient with triphalangeal thumb-polysyndactyly syndrome combined with congenital heart disease and optic disc coloboma: a case report

**DOI:** 10.1186/s12920-020-00821-x

**Published:** 2020-11-20

**Authors:** Anna Zlotina, Olesia Melnik, Yulia Fomicheva, Rostislav Skitchenko, Alexey Sergushichev, Elena Shagimardanova, Oleg Gusev, Guzel Gazizova, Tatiana Loevets, Tatiana Vershinina, Ivan Kozyrev, Mikhail Gordeev, Elena Vasichkina, Tatiana Pervunina, Anna Kostareva

**Affiliations:** 1 Almazov National Medical Research Centre, 2, Akkuratova Street, Saint Petersburg, Russia 197341; 2 Computer Technologies Laboratory, ITMO University, Saint Petersburg, Russia; 3 Institute of Fundamental Medicine and Biology, Kazan Federal University, Kazan, Russia 420008; 4 RIKEN Cluster for Science, Technology and Innovation Hub, RIKEN, Yokohama, 230-0045 Japan; 5 Department of Women’s and Children’s Health, Center for Molecular Medicine, Karolinska Institute, 17176 Stockholm, Sweden

**Keywords:** Triphalangeal thumb-polysyndactyly syndrome, DORV, Microphthalmia with coloboma, 7q36 duplication, *LMBR1*, Long-range sonic hedgehog (SHH) regulator (ZRS)

## Abstract

**Background:**

Triphalangeal thumb-polysyndactyly syndrome (TPT-PS) is a rare well-defined autosomal dominant disorder characterized by long thumbs with three phalanges combined with pre- and postaxial polydactyly/syndactyly of limbs. By now, the syndrome has been reported in several large families from different ethnic backgrounds, with a high degree of inter- and intrafamilial variability. The genome locus responsible for TPT-PS has been mapped to the 7q36.3 region harboring a long-range sonic hedgehog (SHH) regulatory sequence (ZRS). Both single-nucleotide variants and complete duplications of ZRS were shown to cause TPT-PS and similar limb phenotypes. TPT-PS usually forms as isolated limb pathology not associated with additional malformations, in particular, with cardiovascular abnormalities.

**Case presentation:**

Here we report on a rare Russian neonatal case of TPT-PS combined with severe congenital heart disease, namely double outlet right ventricle, and microphthalmia with optic disc coloboma. Pedigree analysis revealed TPT-PS of various expressivity in 10 family members throughout five generations, while the cardiac defect and the eye pathology were detected only in the proband. To extend the knowledge on genotype–phenotype spectrum of TPT-PS, the careful clinical and genomic analysis of the family was performed. High-resolution array-based comparative genomic hybridization (array-CGH) revealed a ~ 300 kb microduplication of 7q36.3 locus (arr[GRCh37] 7q36.3(156385810_156684811) × 3) that co-segregated with TPT-PS in the proband and her mother. The duplication encompassed three genes including *LMBR1*, the intron 5 of which is known to harbor ZRS. Based on whole-exome sequencing data, no additional pathogenic mutations or variants of uncertain clinical significance were found in morbid cardiac genes or genes associated with a microphthalmia/anophthalmia/coloboma spectrum of ocular malformations.

**Conclusions:**

The results support the previous data, indicating that complete ZRS duplication underlies TPT-PS, and suggest a broader phenotypic impact of the 7q36.3 microduplication. Potential involvement of the 7q36.3 microduplication in the patient’s cardiac and eye malformations is discussed. However, the contribution of some additional genetic/epigenetic factors to the complex patient`s phenotype cannot be excluded entirely. Further comprehensive functional studies are needed to prove the possible involvement of the 7q36.3 locus in congenital heart disease and eye pathology.

## Background

Triphalangeal thumb-polysyndactyly syndrome (TPT-PS, OMIM #174500) represents a rare congenital autosomal dominant disorder usually characterized by long thumbs with three phalanges combined with pre- and postaxial polydactyly and syndactyly of limbs. By now, the syndrome has been described in several families belonging to different ethnic groups [1–7]. Using linkage analysis, the genetic locus responsible for TPS-PS was mapped to chromosome 7q36 [2–4]. The same locus was found to be associated with other limb malformations such as preaxial polydactyly, complex polysyndactyly, syndactyly type IV, Laurin-Sandrow syndrome and acheiropodia [8–13]. The 7q36.3 chromosomal region encompasses several annotated genes, including *LMBR1.* The intron 5 of *LMBR1* was shown to harbor an evolutionary conserved element ZRS (ZPA regulatory sequence) representing a long-range enhancer of *SHH* gene [14]. The Sonic hedgehog (SHH) is known as a key morphogen involved in organogenesis. Being expressed in the zone of polarizing activity (ZPA) of a developing limb bud, SHH regulates patterning and morphogenesis of extremities. In particular, specific spatiotemporal SHH expression in ZPA defines an anterior–posterior axis of the limbs, thus ensuring the correct number and morphology of the digits [15]. It was shown that point mutations in ZRS and pre-ZRS (a noncoding sequence 500 bp upstream of the ZRS) can lead to TPT-PS [7, 16–19]. Besides, ZRS genomic duplications also proved to be associated with similar clinical phenotypes [5, 6].

While the triphalangeal thumb can be part of some complex syndromic conditions (e.g. Holt-Oram syndrome, lacrimo-auriculo-dento-digital syndrome, Duane-Radial Ray syndrome), TPT-PS usually forms as an isolated limb pathology not combined with additional malformations. In particular, congenital heart defects (CHDs) are not typical for patients with TPT-PS. Here we present a rare case of asymmetrical triphalangeal thumb-polysyndactyly syndrome combined with a severe CHD, namely double outlet right ventricle (DORV), and microphthalmia with optic disc coloboma. To get new insights into the genotype–phenotype spectrum of TPT-PS and molecular mechanisms underlying the syndrome, we performed the careful clinical and genomic analysis of the family. According to the pedigree analysis, TPT-PS of various expressivity was followed in 10 family members throughout five generations, while the heart defect and the eye pathology were detected only in the proband. High-resolution array-based comparative genomic hybridization (array-CGH) revealed a 299 kb microduplication of 7q36.3 chromosomal region encompassing the ZRS *cis*-regulatory element of *SHH* that co-segregated with TPT-PS in the family. Using whole exome sequencing (WES), no additional point pathogenic or likely-pathogenic variants were detected in the genes associated with the heart-eye phenotype. The data obtained confirm a role of complete ZRS duplication in TPT-PS pathogenesis and extend our knowledge on a phenotypic impact of the 7q36.3 copy number gain. Potential involvement of the microduplication in the patient’s cardiac and eye malformations is discussed.

## Case presentation

### Clinical description of the family

**Fig. 1:**
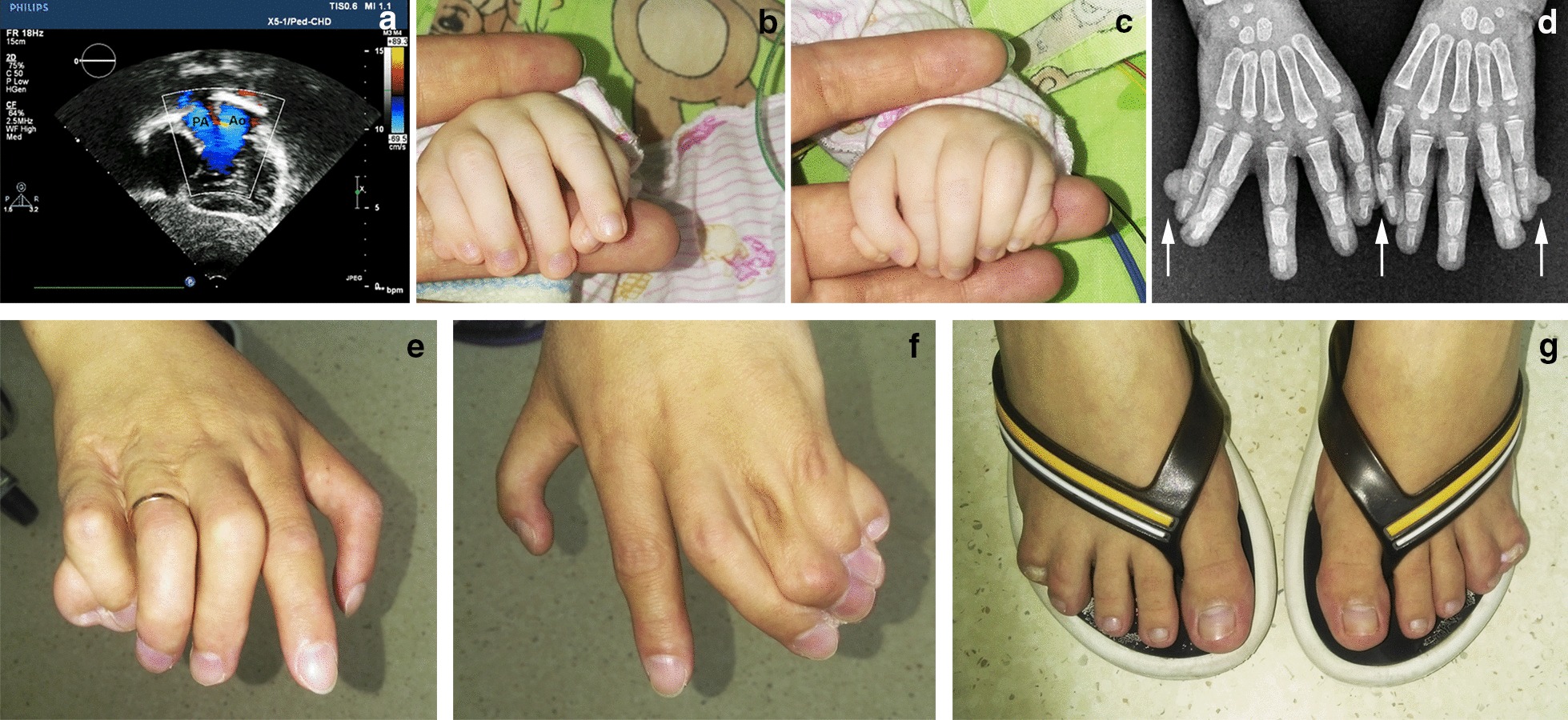
Clinical features.** a** Echocardiogram of the patient indicating double outlet right ventricle. PA—pulmonary artery, Ao—aorta. **b**–**d** Right and left hands of the proband with TPT-PS. Note bilateral triphalangeal thumb with asymmetrical pre- and post-axial polydactyly (arrows) and skin syndactyly involving fingers IV and V. **e**–**g** Hands and feet of the proband`s mother. Note triphalangeal thumb, post-axial polydactyly with total skin syndactyly of fingers III-V as well as syndactyly of fingers IV/V on the feet

The proband, a two-month-old girl, was born as the second child of healthy non-consanguineous parents of Russian origin. The couple has an elder healthy son; another pregnancy ended in a miscarriage. During the 22–23 week of gestation, multiple congenital malformations were detected in the proband including a heart defect (double outlet right ventricle with pulmonary stenosis (Fig. 1a)), deformities of hands (post-axial polydactyly) and a single umbilical artery.

At birth, baby`s weight was 3150 g, body length—51 cm, head circumference—35 cm, chest circumference-33 cm. Routine clinical examination confirmed the limb pathology that included bilateral triphalangeal thumb with asymmetrical pre- and post-axial polydactyly and skin syndactyly of fingers IV/V (Fig. 1b–d). Lower limbs were not affected. Ophthalmological assessment revealed microphthalmia with severe coloboma involving the choroid, retina and the optic disc. In addition to physical malformations, the patient demonstrated psychomotor delay (MQ = 0.39; DQ = 0.5). The radical surgical correction of the heart defect was successfully carried out in two steps within the first year of life.

**Fig. 2:**
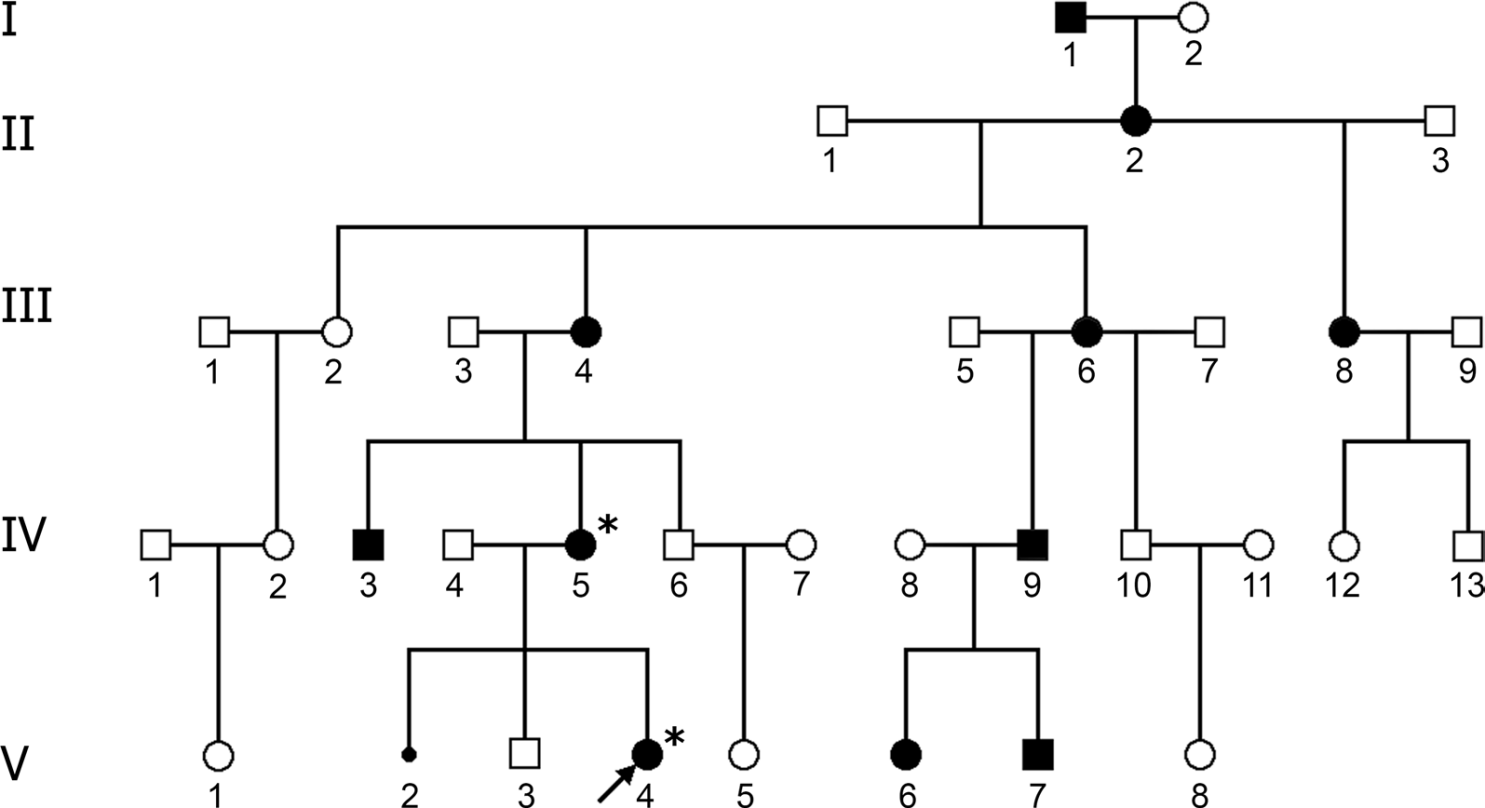
Pedigree of the described family with TPT-PS. TPT-PS-affected family members are marked in black based on clinical description and family history. The arrow points to the proband with TPT-PS combined with congenital heart defect and microphthalmia with coloboma (V-4). Spontaneous abortion of the proband's mother is shown by a small circle (V-2). Asterisks mark the individuals with performed genetic test

According to the medical history, as many as 10 family members presented with TPT-PS throughout 5 generations from the maternal side (Fig. 2). Notably, a high degree of clinical heterogeneity was noted with the most severe phenotype being detected in the proband`s mother (IV-5). In particular, along with triphalangeal thumbs she demonstrated post-axial polydactyly with total skin syndactyly of fingers III-V of the hands as well as syndactyly of fingers IV/V of the feet (Fig. 1e–g). The proband`s grandmother (III-4) presented with hand pre-axial polydactyly with syndactyly of fingers III-V; family members III-6 and III-8 had multiple syndactyly; II-2—syndactyly of fingers IV and V; I-1 showed hand post-axial polydactyly with syndactyly of fingers IV and V. The limb malformations of the rest affected family members were also represented by triphalangeal thumb but could not be specified in details.

### Genomic findings

For standard karyotyping, routine cytogenetic analysis of GTG-banded metaphase chromosomes was performed on phytohaemagglutinin (PHA)-stimulated peripheral blood lymphocytes. Standard cytogenetic analysis of the proband showed a normal female karyotype (46, XX), which excluded numerical or large-scale structural chromosomal rearrangements as a cause of the complex phenotype. Besides, bidirectional target Sanger sequencing did not detect any pathogenic point mutations in intron 5 sequence of *LMBR1* (ID 64327) as well as in protein-coding regions of *NKX2.5* (ID1482) and *TBX5* (ID6910) genes earlier reported to be associated with triphalangeal thumb, structural heart defects, or combined heart-hand pathology such as Holt-Oram syndrome, respectively.

**Fig. 3:**
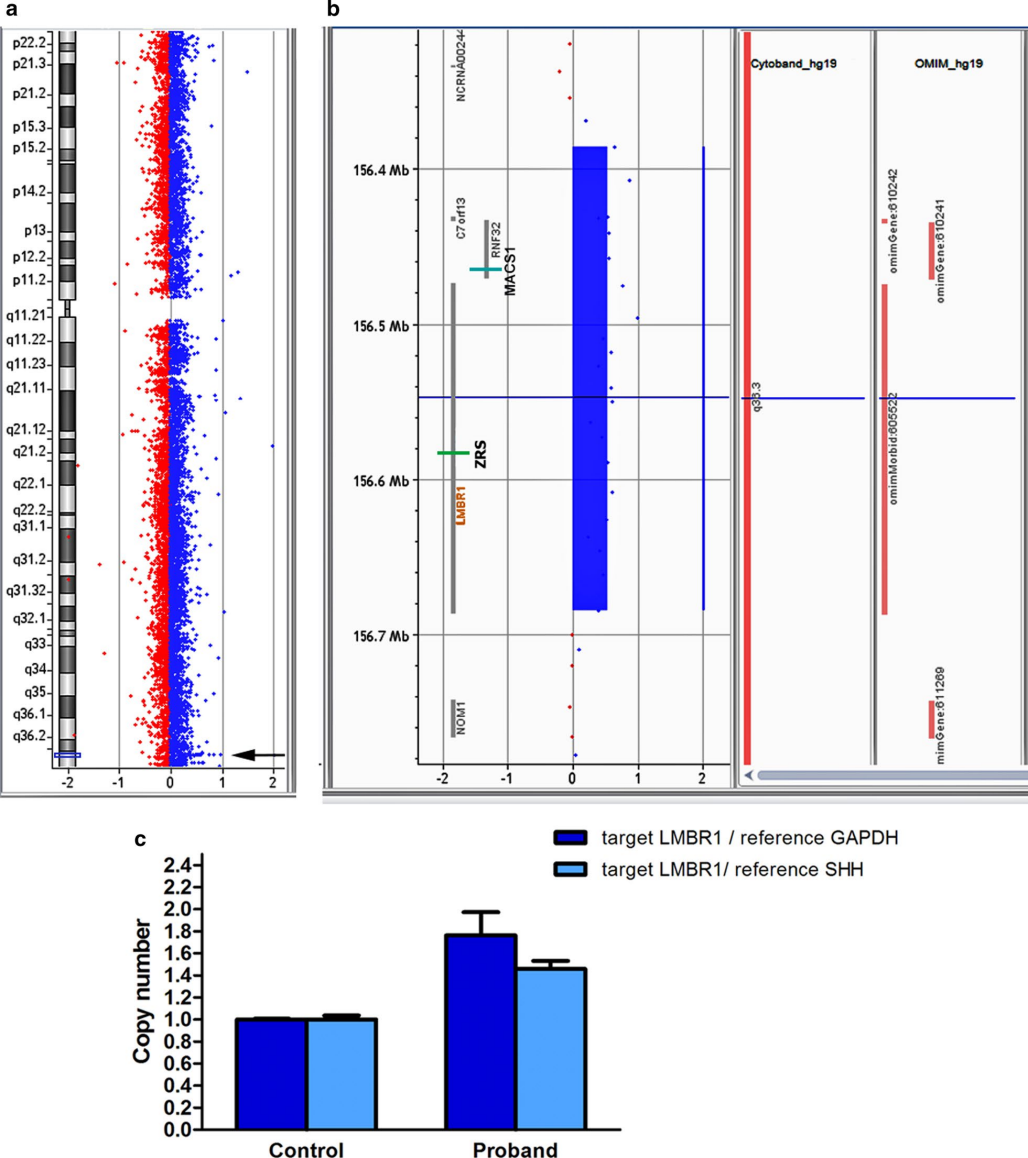
Detection of 7q36.3 duplication. **a**, **b** High-resolution cytogenomic analysis of the proband using comparative genomic hybridization on Agilent 180 K microarray. **a** The whole view of chromosome 7. The microduplication is shown by an arrow. **b** The enlarged 7q36.3 region with imported tracks of gene annotations. The data illustrate the presence of a ~ 300 kb microduplication (arr[GRCh37] 7q36.3(156385810_156684811) × 3) encompassing *LMBR1*, *RNF32* and *C7orf13*. ZRS and MACS1 enhancers of *SHH* are depicted as coloured bars. **c** Confirmation of 7q36.3 duplication using quantitative real-time PCR analysis (qPCR). qPCR data revealed three copies of the *LMBR1* gene in a patient DNA sample as compared to two copies of the gene in a control DNA sample. The data were normalized against *GAPDH* and *SHH* genes using the comparative ΔΔCt method

To screen for copy number variants (CNVs), oligonucleotide array-based comparative genomic hybridization (array-CGH) was carried out. As a platform, Agilent 180 K array with median probe spacing 13 kb was used (SurePrint G3 Human CGH Microarray, Agilent Technologies, Santa Clara, CA, USA). The sample preparation and hybridization procedure were performed following the manufacturer’s recommendations. The data obtained were processed and analyzed using Feature extraction (v9.5) and CytoGenomics (v3.0.1.1) Softwares (Agilent Technologies). CNVs (gains and losses) were called using an aberration detection statistical algorithm ADM-2, with a sensitivity threshold of 6.0. Clinical significance of the findings was evaluated based on the data from publically available databases of normal and pathogenic genome variants, including Database of Genomic Variants (DGV), Online Mendelian Inheritance in Man database (OMIM), Database of Chromosomal Imbalance and Phenotype in Humans using Ensembl Resources (DECIPHER). Array-CGH revealed an interstitial microduplication spanning 299 kb region at 7q36.3 cytoband (arr[GRCh37] 7q36.3(156385810_156684811) × 3) that encompassed three annotated genes (*C7orf13, RNF32* and *LMBR1*) (Fig. 3a, b). The gain of 7q36.3 region was confirmed by quantitative polymerase chain reaction (qPCR) using SYBR Green Master Mix (Evrogen, Russia) (Fig. 3c). The assay was performed for *LMBR1* gene sequence (RefSeqGene NG_009240.2). The quantity assessment of the target sequence was carried out relative to a normal control DNA. The relative copy number was evaluated using the comparative ΔΔCt method with *GAPDH* and *SHH* genes being used for normalization. The qPCR primers were the following: F 5′-ATGCTTTGTGCGGGAAATCCA-3′, R 5′- GTCTCCTCCCTCCTGAATCCAT-3′ (*LMBR1*); F 5′- CTTAAAAAGTGCAGGGTCTGGC-3′, R 5′-TGCTGTAGCCAAATTCGTTGTC-3′ (*GAPDH*); F 5′- TGTTTGCTCTTCGGGCAGAT-3′, R 5′-CGTCTGTTACCGTCCTCACC-3′ (*SHH*). The samples were run in triplicate and the data were analyzed and illustrated in the GraphPad Prism software. The duplication was seen as a ~ 1.5 fold relative copy number. The CNV was submitted to the European Variation Archive (EMBL-EBI, https://www.ebi.ac.uk/eva/), accession PRJEB32845. The CGH and qPCR analysis of the probandʼs mother detected the same microduplication (the data not shown). No additional clinically-relevant copy number variants were identified in the proband and her mother. DNA samples from other relatives were not available for genetic analysis.

To exclude the possibility of presence of additional morbid loci underlying the cardiac and eye pathologies in the proband`s clinical phenotype, high-throughput whole exome sequencing was performed. DNA-library was prepared using the SureSelectXT Human All Exon v6 target enrichment kit (Agilent Technologies, Santa Clara, CA, United States), design of which deeply covers protein-coding regions of the genome. Sequencing run was carried out with the Illumina HiSeq4000 genome analyzer (Illumina, San Diego, CA, United States). Average target region coverage was ∼ × 150 with 95% of the target regions being covered to a depth of 20 or more. The data was processed and analyzed according to a workflow earlier described [20, 21]. In brief, alignment was performed using Burrows-Wheeler Aligner (BWA-MEM-0.7.1) with GRCh37/hg19 genome assembly as a reference. The data processing and variant calling was performed according to Broad institute GATK Best Practice. Genomic variants were annotated using Annovar [22]. Population frequencies and clinical significance of the variants were evaluated based on Genome Aggregation Database (gnomAD) resources and according to ACMG guidelines [23]; the functional prediction was made based on dbNSFP (v3.3a). Expression rank in the heart tissue (GTEx dataset) was used as an additional characteristic for variant evaluation.

The called variants were filtered according to their exonic function and population frequencies so that only rare protein-changing variants such as missense, nonsense, frameshifts or splicing variants with allele frequency < 0.001 were selected for further analysis (Additional file 1). Then, the variants were evaluated based on gene functions, clinical annotations, and prediction of variant functional effect. Special attention was paid to genes known to be associated with isolated CHDs, microphthalmia/anophthalmia/coloboma (MAC) spectrum of ocular malformations or syndromic forms that combine cardiac and eye pathologies. Neither pathogenic or likely pathogenic variants nor variants of uncertain clinical significance (VUS) were found in well-known morbid cardiac genes including DORV-associated loci such as *NKX2-5, ZFPM2/FOG2, TBX5, NODAL, CFC1, GDF1, TDF1, ACVR2B, ZIC3, NPHP4*. Besides, no possible candidate genes that are highly expressed in the heart tissue and involved in cardiogenesis or myocardial functioning were detected as well. Further review of the gene list did not reveal pathogenic/likely pathogenic variants or VUS in the genes commonly associated with isolated or syndromic MAC including *VSX2, RAX, GDF6, BCOR, CHD7, HCCS, SHH, SOX2, OTX2, PAX6, STRA6, ALDH1A3, RARB, FOXE3, BMP4, BMP7, ATOH7, C12orf57, TENM3 (ODZ3), VAX1, SALL2, YAP1, GDF3, ABCB6, RARB, SMOC1, TFAP2A* [24, 25]. Besides, we looked attentively at the *LMBR1*, *C7orf13* and *RNF32* genes located at the 7q36.3 duplicated genomic segment. Such analysis did not reveal any potential modifier variants that could contribute to the severe clinical picture of the proband. Thus, no genomic variants of interest that could explain the particular cardio-eye phenotype of the patient were identified.

## Discussion and conclusions

The present study reports on a new familial case of the triphalangeal thumb-polysyndactyly syndrome. Earlier the syndrome was described in several distinct ethnic groups including Dutch, Indian, Turkish, Chinese and Germany populations [2–7]. To the best of our knowledge, here we provide the first comprehensive phenotype-genotype characterization of a large family of Russian origin with TPT-PS. The affected family members demonstrated variable clinical expression, which conforms to the previous data indicating a high degree of inter- and intra-familial phenotypic variability and progression through generations in TPT-PS pedigrees [3, 4, 7].

Using high-resolution array-CGH, we identified a 299-kb microduplication (7q36.3) that co-segregated with TPT-PS phenotype in the proband and her mother. This CNV encompassed the *LMBR1* locus comprising the ZPA regulatory sequence (ZRS), which lies ~ 1 Mb upstream from the target gene *SHH*. By this moment, several overlapping microduplications of different size ranging from 78- to 588-kb have been identified in association with TPT-PS [4–6, 12, 26, 27]. While point mutations in the ZRS are shown to provoke ectopic SHH-expression on the anterior margin of the limb bud thus leading to TPT-PS [28], the precise molecular mechanism by which ZRS duplications cause severe limb phenotypes is still not deciphered. It is suggested that ZRS duplications may result in an increased number of binding sites for regulatory proteins leading to *SHH* over- or misexpression [5, 13]. Alternatively, they can interfere with a proper long-range *SHH* regulation by disruption of boundaries of the SHH-ZRS topological domain of chromatin [13, 19, 29].

Notably, the 7q36.3 microduplications have been primarily reported in isolated cases of TPT-PS not associated with additional congenital malformations. Here we described the patient who presented a complex combination of TPT-PS with a severe congenital heart defect and ophthalmological dysmorphia. The data obtained could be an evidence of a broader clinical spectrum caused by copy number gain of *SHH* regulatory sequence. In support of this hypothesis, a vital role of sonic hedgehog signaling was shown for the heart and eye development [30–36]. From clinical experience, partial trisomy for the region 7q36.3- > qter (karyotype: 46,XY,der(2) t(2;7)(q37.2;q36.3)) was described in a boy with a unique combination of congenital malformations that included skin syndactyly of the hands and bilateral postaxial polydactyly, transposition of the great arteries with ventricular and atrial septal defects, and anterior chamber eye anomalies [37]. While some phenotypic impact of the partial 2q37.2qter monosomy can not be excluded, 7q36.3 copy number gain seemed to contribute to the complex clinical picture of the patient. Besides, a 7q terminal microdeletion encompassing *SHH* was detected in a child with a complex phenotype including a papilla coloboma [38]. Finally, heterozygous *SHH* mutations are known to be a cause of isolated microphthalmia with coloboma 5 (MIM#611638). In particular, a heterozygous 24-bp deletion in the 3`-end of *SHH* coding region was revealed in a non-syndromic case of colobomatous microphthalmia [39]. Schimmenti and co-authors suggested that the intergenic deletion could interfere with the autocatalytic cleavage of a SHH molecule into N- and C- terminus. According to the authors, ocular malformations might be caused by decreasing the dosage of N-terminus of SHH in the developing eye [39]. Previously, a negative phenotypic effect of both the decreased expression and over-expression of Shh was noted during ocular morphogenesis in zebrafish and chicken [40, 41]. It was also suggested that misexpression of *SHH* could severely affect the downstream ocular genes such as *PAX2* [39].

The molecular-cellular mechanisms by which the 7q36.3 microduplication could cause the ocular and cardiac phenotype in the reported patient are obscure. It might be hypothesized that the duplication could disrupt the proper *SHH* expression during the heart and eye development. By now, several *cis*-regulatory elements responsible for embryonic *Shh* expression within the brain, notochord, pharyngeal endoderm, laryngotracheal tube, lung buds, gut and limb buds were uncovered across a 1 Mb-region upstream of *Shh* in different animals [29, 42]. However, it should be noted that the majority of known *Shh* enhancers are not located within the genomic region involved in the duplication [29, 42]. Apart from ZRS, this region harbors another well-characterized regulatory element MACS1 that ensures widespread Shh expression in the epithelium of the gut, stomach, lungs and laryngotracheal tube [29]. Keeping in mind that ZRS has been characterized exclusively as a limb-specific *SHH* enhancer and that MACS1 is rather a gut-specific element, it could be hypothesized that the 7q36.3 microduplication may involve additional regulatory elements responsible for proper *SHH* expression during cardiogenesis and ocular morphogenesis.

The revealed microduplication did not cause any evident cardio- or eye-phenotype in other TPT-PS-affected relatives. It raises the possibility that some additional genetic variants or an epigenetic defect could contribute to the patient`s complex phenotype. That is, a similar case of TPT-PS combined with Tetralogy of Fallot proved to be a consequence of two distinct genetic defects [43]. In particular, the authors demonstrated that the limb pathology was caused by a ~ 288.5 kb microduplication of the 7q36.3 region, while the CHD—by a classical 22q11.21 microdeletion commonly associated with DiGeorge/velocardiofacial syndromes or isolated conotruncal heart defects [43]. Based on results of array-CGH and high-throughput WES, we did not identify any additional structural variations or single-nucleotide variants in ClinVar/OMIM genes shown to be responsible for cardiac defects and microphthalmia/anophthalmia/coloboma spectrum of ocular malformations. It implies that the cardiac-eye component of the patient`s phenotype does not fit to any well-established genetic syndrome such as CHARGE, Cat eye, Oculofaciocardiodental syndromes and others. At the same time, some causative genome variations in the regulatory elements (promoters, deep intronic splice sites, and others) that are not covered by the exome target enrichment system could not be completely excluded.

In conclusion, the present study reports on a new familial case of rare triphalangeal thumb-polysyndactyly syndrome. For the first time we describe a patient with TPT-PS combined with DORV and optic disc coloboma. We support the earlier data, indicating that a complete duplication of the long-range *SHH* limb regulator ZRS underlies the pathogenesis of TPT-PS. Further comprehensive functional studies are required to prove possible involvement of the 7q36.3 microduplication in congenital heart disease and eye pathology. The results obtained confirm the utility of high-resolution array-CGH analysis for establishment of genotype–phenotype correlation in TPT-PS cases. The data are helpful for accurate individual diagnosis and genetic counseling of the affected families.

## Supplementary information

**Additional file 1.** Rare protein-changing genetic variants identified in the proband by whole exome sequencing. The table provides information on missense, nonsense, frameshift and splicing genetic variants with gnomAD exome allele frequency < 0.001. The data on the rare variants include genomic coordinates, “rs” ID, homo-/heterozygosity state, allele frequencies based on gnomAD and Kaviar, gene description and tissue specificity including cardiac and muscle specific expression (GTEx), associated clinical conditions, CADD and other predictors of variant functional effect.

## Data Availability

The data on array-CGH analysis generated and analyzed during the current study are available in the European Variation Archive repository (Accessions: Project # PRJEB32845, Analyses # ERZ965213, https://www.ebi.ac.uk/eva/?eva-study=PRJEB32845). The whole-exome sequencing data analyzed during this study are included in this published article and its supplementary information file.

## Abbreviations

Array-CGH: Array-based comparative genomic hybridization
CHD: Congenital heart defect
CNV: Copy number variant
DECIPHER: Database of chromosomal imbalance and phenotype in humans using ensembl resources
DGV: Database of genomic variants
DORV: Double outlet right ventricle
MAC: Microphthalmia/anophthalmia/coloboma
OMIM: Online mendelian inheritance in man database
TPT-PS: Triphalangeal thumb-polysyndactyly syndrome
VUS: Variants of uncertain clinical significance
ZPA: Zone of polarizing activity
ZRS: Long-range sonic hedgehog regulator
